# Neuromodulation of safety and surprise in the early stages of infant development: affective homeostatic regulation in bodily and mental functions

**DOI:** 10.3389/fpsyg.2024.1395247

**Published:** 2024-06-06

**Authors:** Andrea Clarici, Matteo Bulfon, Yvonne Radin, Jaak Panksepp

**Affiliations:** ^1^Department of Medical, Surgical and Health Sciences, Cattinara Hospital, University of Trieste, Trieste, Italy; ^2^Department of Pediatrics, Institute for Maternal and Child Health, IRCCS “Burlo Garofolo”, Trieste, Italy; ^3^College of Veterinary Medicine, Washington State University, Pullman, WA, United States

**Keywords:** neuromodulators of depression, neurofunctional disorders, internal and external safety, interoception, predictive coding, free energy principle, mother–infant relationship, infant development

## Abstract

Developing a sense of internal safety and security depends mainly on others: numerous neuromodulators play a significant role in the homeostatic process, regulating the importance of proximity to a caregiver and experiencing feelings that enable us to regulate our interdependence with our conspecifics since birth. This array of neurofunctional structures have been called the SEPARATION DISTRESS system (now more commonly known as the PANIC/ GRIEF system). This emotional system is mainly involved in the production of depressive symptoms. The disruption of this essential emotional balance leads to the onset of feelings of panic followed by depression. We will focus on the neuropeptides that play a crucial role in social approach behavior in mammals, which enhance prosocial behavior and facilitate the consolidation of social bonds. We propose that most prosocial behaviors are regulated through the specific neuromodulators acting on salient intersubjective stimuli, reflecting an increased sense of inner confidence (safety) in social relationships. This review considers the neurofunctional link between the feelings that may ultimately be at the base of a sense of *inner safety* and the central neuromodulatory systems. This link may shed light on the clinical implications for the development of early mother-infant bonding and the depressive clinical consequences when this bond is disrupted, such as in post-partum depression, depressive feelings connected to, addiction, neurofunctional disorders, and psychological trauma.

## Early neuroregulations of the relational world of the infant

Adaptation is the main task of the brain and mental apparatus: it requires mainly internal adjustments to novel conditions and aims to maintain the self-organization of any living being. There are today a compelling and growing body of evidence (Beauregard, 2007) that suggest the view that the *subjective nature* and the intentional emotional content of mental processes (e.g., thoughts, feelings, beliefs, volition, all experienced from the “first-person perspective”) significantly influence the various levels of brain functioning (e.g., molecular, cellular, neural circuit) and brain plasticity. These findings indicate that subjective variables must be seriously considered to reach a correct understanding of the neural bases of behavior in humans.

Below, we first illustrate the theory of emotional homeostasis from a particular neuroscientific perspective. In the next section, we describe the one supported by psychoanalysis. In both descriptions, the base regulatory principle, as we will show, is focused on the homeostatic maintenance of a maximum rate of an inner sense of safety (given by the absence of any drive or affect); in other terms, the minimization of inner free energy (given when all the subject’s needs and expectancies are met). In joining these two views, this paper aims to offer an integrated neuropsychoanalytic account.

## Psychoanalytic view on the development of a sense of safety

Psychoanalysis may be defined as the discipline that studies the laws underlying human subjective life, i.e., this discipline’s scientific purposes concern intrapsychic processes. Neuroscientists tend to avoid tackling these questions, considering them as not belonging to their methodological field (e.g., Kinsbourne, 2001). Even many psychoanalysts believe the knowledge of neuroscience as irrelevant to the psychoanalytic scientific endeavor (Blass and Carmeli, 2007, 2015; Michels, 2010). Still, most psychoanalysts would assume that their discipline’s assumptions on mental processes will have to be confirmed, or at least paralleled by comparison, with the other scientific branch that deals with brain processes (for a review, see Canestri, 2015; Yovell et al., 2015).

Therefore, in our discussion of the neuromodulatory aspects of the primary regulatory principle of safety, Joffe and Sandler (1967), for example, showed that psychoanalytic psychology is a psychology of adaptation, dealing in particular with the subjective adaptations to changes in feeling states. This model implies that even the most refined and abstract cognitive representations may be activated only if feelings are associated with them. Specifically, a primary task of the mental apparatus during its maturation and development is to manage feelings: the prominent role of learning from experience is to widen the tolerable range of (conscious or unconscious) experiences without disrupting the fundamental feeling *tone* of safety (inner sense of mastery over one’s feelings, security, and absence of external threatening situations) and well-being (lack of anxiety and distress). Therefore, the neurological and mental apparatuses are constantly concerned with maintaining feelings of self-preservation (i.e., safety), largely maintained by monitoring perceptual constancy and congruence. The successful performance of such an act of integration is accompanied by a definite feeling tone, a fundamental *ego tone*, as Sandler (1960) puts it, that might be figured as a constant background state of mastery over unpleasant feelings, even though such activities often occur outside our usual sphere of awareness. Safety seeking is a fundamental survival skill that enables organisms to actively search for and identify potential sources of safety within their environment. This adaptive ability not only involves the detection and recognition of available resources but also entails the utilization of these resources to address pressing inner needs. By leveraging their innate safety seeking abilities, organisms can effectively navigate their environment to ensure their safety and well-being. It yields an internal sense of comfort, a function related to, but distinct, from the well-studied ability to learn about new dangers (FEAR avoidance; Panksepp, 1998; LeDoux, 2000, 2003).

In human clinical settings, the concept of *safety* has been studied by developmental psychologists and psychodynamic psychologists. Several authors (especially attachment theorists, and psychoanalysts, as for instance Sandler, 1960; Joffe and Sandler, 1967; Bowlby, 1970, 1988; Ainsworth, 1989; Fonagy, 2018) emphasized the role of safety, security and related feelings as critical concepts in the refined attunement of the interaction between the mother and the infant and, henceforth, in the early development of personality and its psychopathology. The idea of *inner safety* was developed in the ‘60s or ‘70s in different contexts, mainly developmental attachment theorists on one side and psychoanalysts on the other, converging on the child’s early attachment processes. The concept has various meanings in these two contexts, and the term safety is used differently in the two related disciplines. Traditionally, attachment theorists refer more to *secure* attachment (*objective safety*), meaning the child’s experience with its mother in the history of their mutual relationship, as it may be objectified by an external observer (Belsky, 1999; Fonagy, 2018). In psychoanalysis (see for example Sandler, 1960; Winnicott, 1965) the same concept is applied, but to emphasize *subjective safety*, an intrapsychic condition achieved through the conscious and unconscious elaboration of affective drives. Unconscious motivational processes (from basic needs to conflictual urges) *aim at the perception* (see the concept of identity of perception and transference; Sandler, 1976), a re-representation of early relational significant and reassuring experiences of the child with its caregivers.

According to attachment theorists (Bowlby, 1970; Ainsworth, 1989), security is the core regulatory process promotes healthy prosocial psychological development from infancy. Bowlby (1988) developmental notion of a “secure attachment” and a “secure base” were implicitly based on a conception of safety–related processes. Still, differently from the psychoanalytic use of the concept, the dissimilarity may be found in the diverse possible origins of the threat (external for attachment theorists, internal for psychoanalysis). Psychoanalysts (Sandler and Joffe, 1968; Fonagy, 2005) stress the importance of intrapsychic adaptation, avoidance of feelings of destabilization, and the development and consolidation of internal *working models* (representations of the caregiver in a specific relation with the subject) to achieve a sense of safety. Attachment theorists *and* psychoanalysts agree that security and safety are based on innate functions that operate soon after birth and govern long-term psychological development and emotional responsivity. Thus, both attachment theory and psychoanalytic models emphasize the primary status and biological function of intimate emotional bonds between individuals: we want to add to the discussion that the maintaining of these emotional intersubjective bonds is controlled by specific neurobiological systems situated within the deeper parts of central nervous system (CNS). Parent-infant affective exchanges rely on the activation of ancient and deep evolutionarily preserved brainstem networks: the infant’s separation distress behavior is mediated by the PANIC/GRIEF system, which promotes reunion with the caregiver, while the complementary parental CARE system (Panksepp, 1998) processes feeling of tenderness: both sustain early attachment development. These basic emotional systems are modulated by various neuroamines and neuropeptides that converge on opioid-mediated processes (Panksepp et al., 1980). The positive interactions between parent and infant facilitate the development of a positive working model of self-other relationships (or internal representations of interactions that have been generalized in an implicit or explicit memory process; Stern, 2014).

Moreover, Panksepp (2010) conceptualized an intermediate layer in the learning processes during the developmental age in the child: he termed this level *the secondary process*. At this level, there is the consolidation in a procedural form of the relationships to attachment figures (introjection; Olds, 2012). These secondary processes take the form of relational implicit memories that have a vital role in the developmental interplay between the basic emotional systems (primary process) and higher cognitive functions (tertiary processes) (see also Solms and Panksepp, 2012). This hierarchical model evolved in the brain to sustain its fundamental principle of minimizing disorganization (or free energy; see Friston et al., 2006), warranting the organism to remain in the vital homeostatic bounds of inner safety.

## The neuropsychology of specific safety signals

The neurobiology of processes associated with the affective states related to safety has only recently received experimental neuropsychological attention (Rogan et al., 2005). These researchers have tried to identify and *localize* neural networks connected to conditions of safety and security, not simply to be considered as states of lack of fear of dangers but as the result of specific comforting signals, which depart from subcortical motivational networks. Other studies (Eisenberger et al., 2011) have tried identifying affective cortico-limbic systems connected to safety modulation. Fear learning and safety-related conditions are undoubtedly interconnected since safety signals reduce conditioned fear responses (Grillon and Ameli, 2001), indicating the existence of an interaction with the neural circuitry underlying fear conditioning, of which the amygdala is a crucial structure (LeDoux, 2000). The critical areas of the safety processing system in the brain appear in specific areas of the *dorsal striatum* (Rogan et al., 2005) and portions of the *ventromedial prefrontal cortex* (Eisenberger et al., 2011). Both animal and human research have shown that the ventromedial prefrontal cortex (VMPFC) is an essential component of neural mechanisms underlying perceived safety in fear learning (Schiller et al., 2008). However, the correlation between animal and human studies is not univocal (Josselyn et al., 2004). A cascade of fear-related brain networks, including the periaqueductal grey, amygdala, and bed nucleus of the stria terminalis, seems to be deactivated in response to safety signals that decrease threat value (Mobbs et al., 2010). Notably, all the structures mentioned above seem to be mainly involved in safety regulation, and they possess a high quantity of oxytocin (OT) receptors on the surface of their neuronal cells (Gimpl and Fahrenholz, 2001). OT seems to modulate the responses to stress and increase the subjective feelings of comfort and safety. Studies on natural variations of oxytocin receptor polymorphism confirm the linkage between OT and feeling of safety. For instance, a specific homozygotic configuration of oxytocin receptor enhances self-confidence and optimism in healthy individuals (Saphire-Bernstein et al., 2011) in conjunction with lower levels of perceived loneliness and stress responses (Lucht et al., 2009) compared to individuals who carry the heterozygote alleles. Positive affectivity, a sense of being in a “safe” social environment, and the related ability to seek social resources are considered protective factors for depressive disorders. However, *the effects of oxytocin appear to be stronger when the baseline condition of the subject is already “safe.”* For instance, Declerck et al. (2010) found that people behave more cooperatively under the influence of oxytocin only when interacting with familiar targets. Thus, future studies should aim to investigate the association between OT polymorphism and responsiveness to OT administration in depressed patients and, eventually, the association between OT and the testing environment.

## Brains and minds as safety-seeking agencies

According to the theory of brain information processing, the brain is an *inferential machine*, meaning a sophisticated device capable of generating and testing hypotheses about the causes of its internal states (of the body and the mind) to self-maintain and perpetuate its primary functional organization over time (Friston, 2005). In a previous paper, one of us (Clarici et al., 2019) focused on how this self-organization follows the evolutionary laws of self-preservation and is fundamentally regulated in organic and psychological terms by achieving a basic safety necessary for the organism’s survival. The underlying principle is the *Free Energy Principle* (Friston et al., 2006). It argues that for an organism to self-preserve, it must first minimize the number of surprising events occurring in time. Thus, *surprise* refers to the quantity of unexpected “informational disorder” or the degree of dissipation of its energy. If this dissipation is too high, the organism will gradually disorganize, cease to function, ultimately disintegrate, and consequently die. In physics (and in particular in information theory), “disorder” in this context is formalized in terms of the degree of this measure of dissipation (or entropy) in the system.^1^ It is, therefore, crucial for every organism to possess specific characteristics that allow constant monitoring of its state and the extent of its disorder and to implement measures capable of correcting any excessive deviation from optimal ranges of homeostatic regulation.^2^ In an attempt to simplify the complexities of the mathematical and theoretical formulations underlying information theory, we can summarize that to minimize the degree of surprise (and thus maximize the system’s inner state of safety and security), the organism must, first and foremost, have (1) *boundaries* that delineate the inside from the outside (a Markov blanket); (2) a *sensory-perceptual apparatus* capable of receiving, encoding, and translating the possible causes of external states into an internal representation (internal states); (3) a *motor apparatus* capable of altering the origin of these possible external causes; and (4) an apparatus capable of recording the outcomes of these “experimental tests” deriving from the interaction of the System with the non-System (the external environment) and updating this internal model, by constantly modifying it to adapt it to its needs or the context. Finally, a self-maintaining system must be capable of (5) assessing the reliability of a specific series of events, enhancing the salience of certain stimuli that are more significant for the organism’s survival and preservation. This assessment of reliance (or *confidence*) (a) in incoming relevant signals (*predictive errors*) or (b) in our internal expectations (*prior predictions*) projected outward is inherently a conscious activity linked to the effects of feelings. First and foremost, the world must be *felt* as we navigate through our senses to perceive it. With each of these five components in place, we can assume that an organism can resist its dissipation according to the functioning of *homeostasis*, surviving for as long as the interaction with its environment maintains its organization in dynamic balance to perpetuate and reproduce its “informational heritage.”

## Neuromodulators and their adaptive role in infant development

Given the regulatory role of safety over anxiety argued by Freud (1938[1940])^3^, we could wonder whether the safety feelings are inversely correlated to *social separation-distress processes* that Panksepp (1998) postulated stemming from what he called the PANIC/GRIEF system or to more general anxiety responses that help the child avoid dangers and external threatening situations (such as the FEAR system). As suggested by Panksepp (2005a,b), the genesis of panic attacks (i.e., the behavioral analog of feeling ultimately *unsafe and* utterly lost) is closely related to our mammalian capacity to have social bonds. As conceptualized initially by Panksepp (1992), oxytocin and the concept of safety are strongly associated. Panksepp states in this respect that the vicissitudes of the perception of the proximity of a caregiver are regulated by various neuromodulators (e.g., oxytocin, together with other neuromodulators such as endogenous opioids and prolactin) that create and maintain a sort of affective background sense of safety and security. The setting of these bounds on the homeostasis of parent-infant attachment and bond is established in the critical first stages of development.

We are herewith reviewing the hypothesis that the neuroanatomical system supporting the achievement of maladjusted attachment styles and those supporting adaptation and mature behavior may be connected to a standard safety and motivational regulatory homeostatic system involving, to some extent, oxytocin. The origin of such feelings seems rooted in the supportive social behaviors of a central attachment figure (or caregiver), for the mother provides the infant with a warm and intimate sense of psychological safety and security (Heinrichs et al., 2009). However, the infant’s sense of inner safety and security constitutes the base for confidence in oneself and trust in others. This sense of confidence is constructed with brain chemistries that carry such experiences of social commitment and support. Very early in life, the infant’s attachment-seeking behaviors come to be directed toward individuals who sustain such rewarding and comforting feelings. The first brain chemistries demonstrated to mediate such effects were endogenous opioids (for reviews, see Panksepp et al., 1980, 1985; Panksepp, 1981), but in the 1990s, great interest was extended to the analysis of oxytocin (OT) in the mediation of social affects and attachments (Panksepp, 1992; Carter, 1998; Uvnäs-Moberg, 1998), and the previous decade observed an expansion of work to humans through the use of intranasal oxytocin, which was found to penetrate the brain (Born et al., 2002). The dopaminergic system and its interactions with oxytocin have been implicated in many aspects of motivational states and affiliative behavior (Leckman et al., 2005). There is a significant anatomical overlap between the oxytocin and dopamine neuron populations. Projections of oxytocinergic neurons in the medial preoptic area to the ventral tegmental area (the origin of the mesocorticolimbic dopamine pathway that mediates behavioral responses to stimuli of salience) are associated with the regulation and maintenance of social bonds, including human parenting and affiliative behavior (Young and Wang, 2004). In this perspective, the critical effect of oxytocin is to promote feelings of safety and, hence, social confidence, which are of foremost importance in affectively positive human relations, from mother–infant bonding to adult attachments and experiences of friendships (Heinrichs et al., 2009). Even though it is also clear that endogenous opioids are very robust mediators of similar psychological processes, we here focus on oxytocin because of the great potential for immediate applications to various human clinical problems ranging in adults (from addiction to depression to suicidality; Stevens et al., 2013; Yovell et al., 2016) and in child development (Quattrocki and Friston, 2014). One point often lost in these discussions is the anatomy of oxytocin systems, concentrated in ancient subcortical “animalian” regions of mammalian brains (Panksepp, 1998). Hence, it is essential to recognize that these emotional foundations of human minds may need to be discussed at many distinct levels of analysis — from primary processes we share with animals to higher-order ones, some of which are unique to humans (for a discussion of this levels of analysis issues, see Panksepp, 2011a,b). Suppose we use terminologies that are generally more critical for human interactions, such as the “trust” needed in financial negotiations. In that case, we may focus on social cognitive processes mediated by oxytocin and be confronted by paradoxical results such as intranasal oxytocin promoting financial “trust” in many humans but reducing it among individuals with borderline personality disorders (Bartz J. et al., 2010). At the same time, both effects may make sense if we understand how higher mental processes might respond to elevations of primary affective processes such as a sense of safety, security, and confidence. Such feelings may ramify widely in higher regions of the brain, leading at times to love, at times to trust, social congeniality, and other good social feelings that require “propositional objects” — namely specific cognitive representations of events or of significant others from which the details of our lives are constructed.

## The particular role of oxytocinergic neuromodulation in constituting prosocial life in the infant

Numerous findings in animals (Boer, 1993; Bales and Carter, 2003) and humans (Kendrick, 2000) have now converged to highlight the role of the evolutionarily ancient neurohormone oxytocin (Gimpl and Fahrenholz, 2001) in promoting parental urges and mediating parent-infant affective exchanges (Pedersen et al., 1982; Panksepp, 1992, 1998; Carter, 1998) in moderating social stress responses (Panksepp, 1992; Heinrichs et al., 2003), and also facilitating adult pair-bonding (Insel and Young, 2001; Domes et al., 2007; Ditzen et al., 2009).

In humans, OT was thought to promote prosocial behaviors: following intranasal OT administration, individuals show enhanced trust (Baumgartner et al., 2008), promote the understanding of others (Domes et al., 2007), and various “compassionate” behaviors (Barraza et al., 2011). These seemingly diverse forms of human prosocial behaviors at their fundamental levels rely on ancient basic emotional subcortical systems and their neurochemistry (Panksepp, 1998). Because of the diversity of behaviors observed, it now seems reasonable to inquire whether a general oxytocin-induced improvement of the “psycho-relational” behaviors that have been studied in humans may share a common denominator. We hypothesize that this basic affective signal of enhanced comfort may be associated with the basic feeling of safety, which opens the door to social confidence (brought about by the absence of feelings of PANIC/GRIEF). One of the most recent theories for how oxytocin modulates behavior is the “social salience” hypothesis. According to Love (2014), many of the several oxytocin-induced changes in mammals’ social behavior may be under control of dopamine reward system. The location of oxytocin receptors in the mesocorticolimbic dopamine system places oxytocin in an ideal position to influence a wide range of motivated behaviors.

As already stated, endogenous opioids operate with other neuropeptide systems like oxytocin to enhance the feeling of comfort and reduce feelings of PANIC/GRIEF. We here develop the idea that OT may contribute to helping mediate feelings of *intrapersonal safety,* which at the psychobehavioral level is expressed as increased confidence, prosocial behaviors and other higher-order cognitive expressions of such primal processes, such as trust, social cognition, empathy, love and compassion (Panksepp, 1992, 2009). Of note, disorders in mother-infant primary interaction and mental pain experienced in Postpartum Depression share standard anatomical and functional neural systems, one of the most studied being the PANIC separation-distress system (Panksepp, 2005a,b). This system also appears to be amply regulated through oxytocinergic pathways. This assumption has implications for the clinical management of social bonding and related disorders in humans and animals (Panksepp, 2005a,b). In diverse human cultures, people talk about losing a loved one in terms of painful feelings. Evidence suggests that localized electrical stimulation of subcortical brain areas involved in regulating pain can provoke separation cries, and endogenous neuroendocrinological substances regulate these responses, indicating an evolutionary relationship between social and physical pain systems. Additional areas of painful feelings related to PANIC/GRIEF include the anterior cingulate, the bed-nucleus of the *stria terminalis*, the ventral septal and dorsal preoptic areas, the dorsomedial thalamus, and the *Periaqueductal Gray* (PAG) of the brainstem (Panksepp, 1998). Notably, most of these areas coincide with the *Central Oxytocinergic System* (see above Gimpl and Fahrenholz, 2001). The effects of OT on the emotional response of PANIC/GRIEF were more robust than on physical pain (Panksepp and Watt, 2011). Along this way, it was also discovered that other important chemistries robustly quelled PANIC/GRIEF, such as endogenous opioids and prolactin, as well as brain chemistries that intensify feelings of distress, such as CRF and glutamate (Panksepp, 2005a,b). A critical question is whether and to what extent OT brain pathways mediate affective and maternal urges.

In line with recent theories of its *modulating* role (instead of *promoting* one), OT does not appear *essential* for mothering: its removal through OT antagonist substances does not interfere with mothering behavior (Nelson and Panksepp, 1998). Oxytocin helps develop a secure attachment by facilitating and extending the security period during bonding formation. Maternal attachment patterns have been found to impact the caregiving practices of mothers significantly. Specifically, mothers with secure attachment patterns during interactions with their infants tend to produce a higher level of oxytocin, known as the “love hormone,” which increases the reward experience. This, in turn, may contribute to the mother’s ability to provide consistent and nurturing care. These findings highlight the importance of maternal attachment patterns in developing healthy mother-infant relationships and may have implications for promoting positive parenting practices (Strathearn et al., 2009).

Nevertheless, OT may not be implicated only in “positive” rewarding or prosocial basic emotional processes. Another hypothesis is that oxytocin may be a neuropeptide involved in the codification and informative function in the CNS of conditions of achieved emotional homeostasis. Incidentally, this may be why OT effects are so subtle and not perceivable even after administration of high doses in the CNS. This passage discusses the hypothesis that homeostatic processes do not inherently promote prosocial or developmental novel states but instead aim to maintain a dynamic stationary state. This suggests that OT may also be involved in psychopathological processes if it is present to maintain a maladaptive state of “well-being,” and its release is linked to any developmental new issue experienced as a disruption. Ultimately, this could inform the psychic apparatus of a potential deviation from the maladaptive but stable state. (Yong, 2012; Miller, 2013).

Intranasal oxytocin has shown an anxiolytic effect, as measured by decreased corticosterone in men during a test involving a stressful public speaking task (Heinrichs et al., 2003). Women viewing pictures of loved ones have high brain activity in dopaminergic pathways associated with reward, which is supposed to be modulated by the highly represented OT and arginine-vasopressin receptors in the same areas (Bartels and Zeki, 2004), as do people who describe themselves as being “intensely in love” (Fisher and Aron, 2005). All these studies confirm a positive effect of OT on prosocial mental processes and a substantial decrement in anxiety signals along with elevated feelings of safety. In women, various studies have been conducted using intravenous infusions and the measurement of plasma levels of OT during labor and postpartum. Such research shows that oxytocin plasma levels are positively correlated with higher self-reported feelings of social attachment (Tops et al., 2007) and also associated with decreased negative feelings (Mezzacappa and Katlin, 2002). Presumably, the OT-induced affective shifts are also manifested and further processed at higher cognitive levels, but these influences can only be inferred from human self-reports.

## Psychopathologies connected to altered neuromodulation

This review will present in the following sections some disorders that appear to be related to disturbances or alterations in the fundamental principle of *inner safety maximization* (Sandler and Joffe, 1969) or *free energy minimization* (as Friston, 2010 puts it). In fact, according to Friston (2023), from a computational psychiatry perspective states:

if psychology — read as belief updating in the brain — can be cast as a computational process of inference, it follows that psychopathology just is false inference. False inference is meant in the usual sense of false positives (i.e., type I errors); namely, inferring something is there when it is not. Cardinal examples here include hallucinations, delusions and other features of reality distortion seen in psychosis. False negatives (i.e., type II errors) mean inferring something is not there when it is; for example, dissociative disorders, neglect syndromes, derealization phenomena, et cetera. Indeed, when one thinks about psychiatric and neurological disorders, most can be framed as false inference. (p. 257)

We will begin with a description of stress-related disturbances, then move on to describe some psychopathologies deeply related to stress, trauma, or relational issues (functional neurological disorders, pathological addictions), and finally, mood disorders deeply interrelated to relational dynamics. Throughout the discussion, relationships with oxytocin will be highlighted.

## Stress-related diseases and neuromodulation of depressive feelings

Research has shown that the hypothalamic–pituitary-adrenocortical (HPA axis) functions differently in individuals with certain psychological and psychiatric conditions, such as depression, anxiety disorders, and PTSD (Baskerville and Douglas, 2010; Walker and McGlone, 2013; Herman et al., 2016). These conditions are often associated with an altered response to stress. The mechanisms behind maladaptive reactions to chronic stress are still being understood. However, the complex relationship between prolonged HPA axis activation and neural pathway activation in the brain is known. In normal conditions, the stress response “[…] represents an integrated reaction to stressors, broadly defined as real or perceived threats to homeostasis or wellbeing.” (Herman et al., 2016). The HPA axis recruits the energies needed to respond to stressors, whether it is a reactive response or an anticipatory response. After the active phase, the HPA axis activity decreases due to a negative feedback mechanism regulated by glucocorticoids. However, if this system is repeatedly or continuously activated, it can lead to various problems. Studies suggest that prolonged or repeated stress during childhood, caused by negative social experiences, abuse, or parental neglect, can result in more prominent reactions to stressors later in life (Walker and McGlone, 2013). However, genetic components shape personal responses to stress by influencing the presence and density of specific receptors, which will define the neurobiological response patterns to stressors in conjunction with early childhood expressions. According to research, individuals who have experienced emotional trauma or have PTSD have significantly lower levels of oxytocin (OT) in their bodies (Donadon et al., 2018). Some studies suggest that oxytocin receptors located in the amygdala and anterior cingulate cortex may play a role in regulating stress responses, as these areas are also involved in the physiology of PTSD (see for example Sack et al., 2017; Takayanagi and Onaka, 2021; Jin et al., 2023).

Oxytocin exerts a significant anti-stress function by suppressing both the secretory and behavioral reactions to stress within the HPA axis due to its actions within the PVN (paraventricular nucleus), which inhibits the activity of corticotrophin-releasing hormone neurons. It seems that oxytocin also has a role in the modulation of anxiety, acting in the diverse regions involved in these disorders, namely the PVN, SON (supraoptic nucleus), and amygdala. In chronic stress, magnocellular oxytocinergic neurons are inhibited by corticosterone, decreasing the amount of central oxytocin and preventing an anxiolytic and anti-stress effect (Di et al., 2005). The anxiolytic effect of oxytocin would be given by an action via serotoninergic pathways, which are also involved in depressive disorders; these findings are inherent in animal models only (Baskerville and Douglas, 2010). The anti-stress effect of oxytocin would also be due, in synergy with dopamine, to the rewarding part of social and addictive behavior. Other evidence from animal models suggests how crucial dopaminergic pathways and the alteration of their plasticity are in determining depressive spectrum disorders (Lamanna et al., 2022). In this regard, the relationships between the dopaminergic system and oxytocin could be significant in treating these disorders.

## Functional neurological disorders and neuromodulation of depressive feelings

The absence of an organic etiopathogenesis characterizes functional neurological disorders. Interpretation of the establishment and development of these disorders is complex given the variety of symptom manifestations and neurological or psychiatric comorbidities Current theoretical systems recognize several possible causes, predisposing factors and triggering events that can be primarily traced back to the individual’s developmental history, i.e., individual and family history of illness, past and present trauma, organic and genetic vulnerabilities, and others (Edwards et al., 2012; Ludwig et al., 2018; Edwards, 2021). The interpretative model we refer to here is based on the Bayesian model of the brain and the role of attentional components (exteroception, interoception, and proprioception) in establishing of the disorder. In particular, the role of emotional activation is relevant, influencing attentional orientation and salience (see Edwards et al., 2012; Solms and Friston, 2018, for an account of attention and affective precision).

The absence of an organic etiopathogenesis characterizes functional neurological disorders. These disorders are difficult to interpret due to various symptom manifestations and neurological or psychiatric comorbidities (Edwards et al., 2012; Espay et al., 2018; Gilmour et al., 2020; Patron et al., 2022). Various theoretical systems acknowledge several possible causes, predisposing factors, and triggering events that can primarily be traced back to the individual’s developmental history (Edwards et al., 2012; Ludwig et al., 2018; Edwards, 2021). This includes their individual and family history of illness, past and present trauma, organic and genetic vulnerabilities, and more. The interpretative model that we are referring to is based on the Bayesian model of the brain and the role of attentional components such as exteroception, interoception, and proprioception in the establishment of the disorder (Edwards et al., 2012; Edwards and Bhatia, 2012; Edwards, 2021). Emotional activation plays a significant role in influencing attentional orientation and salience. The role of attention and affective precision is explained in detail in works by Edwards et al. (2012) and Solms and Friston (2018).

The purpose of this article is not to delve into the details of this model. Instead, we will focus on the potential role of neuromodulators, specifically oxytocin, in certain disorders. Studies have shown that patients with functional neurological disorders (FND) often have comorbid mood disorders such as dysthymia and depression, anxiety disorders, and stress-related problems (Ludwig et al., 2018; Liang et al., 2021). Other studies have found alterations in the limbic-motor axis, with abnormal amygdala activations and a possible suppression effect on unwanted memories, and, more generally, an abnormal emotional activation (Aybek et al., 2014, 2015; Liang et al., 2021). Moreover, patients affected by FND complain of high levels of pain, both acute and chronic (Espay et al., 2018; Gilmour et al., 2020; Liang et al., 2021). There are very few references in the literature regarding FNDs and the efficacy of drug treatments, especially in randomized controlled trials (Perjoc et al., 2023). These aspects suggest an imbalance in the regulation or expression of neurotransmitters that regulate such emotional and stress aspects in the brain. However, the literature has no complete agreement on this (Liang et al., 2021).

It is essential to conduct further investigation to fully comprehend the role of monoamines such as norepinephrine, dopamine, and serotonin. These monoamines are involved in various affective and adaptive mechanisms, including mood regulation, fear management, pain experience, reward mechanisms, motor programming/regulation, and post-traumatic stress disorder (PTSD). It is also crucial to explore the role of oxytocin in modulating specific affects. Additionally, it is necessary to understand any alterations in the hypothalamic–pituitary–adrenal (HPA) axis and the role of corticotropin-releasing hormone (CRH) and cortisol in patients with functional neurological disorder (FND). These mechanisms appear to be closely related to the neurodynamic of oxytocin. The uncertainty regarding the use and effectiveness of pharmacologic treatments in FND leads to a need for more agreement and knowledge among experts. While antidepressants, antipsychotics, and neuroleptics are used for symptom management, their mechanisms of action concerning symptoms are not yet fully understood. Additionally, therapy cannot act on the neural mechanisms of the disease (Gelauff, 2020; Perjoc et al., 2023).

To summarize, there is much more to learn about functional neurological disorders (FNDs). However, recent research suggests that there may be a connection between altered emotional regulation and bodily experience in patients with FNDs. This has led to increased study of the neural systems, such as the motor, limbic, and executive systems, and the associated neuromodulators (Espay et al., 2018; Liang et al., 2021; Perjoc et al., 2023).

## Neuromodulation of functional disorders connected to attachment styles

Further exploration is needed to understand the complex factors contributing to functional symptoms fully. The age-old debate between nature and nurture is relevant in this context, as research by Torgersen (1986) suggests that both genetic and environmental factors influence the inheritance of somatoform disorders within families in a complex interdependence. Although the concordance rates in monozygotic (MZ) twins were higher (29%) compared to dizygotic (DZ) twins (10%), the difference was not statistically significant. This indicates that although genetics may play a role, it is not the only factor in developing of functional neurological disorders. The study acknowledges the limitations imposed by the small sample size and the shared childhood environments, which were found to be correlated, albeit weakly, with the concordance rates for both MZ and DZ twins. This nuanced interpretation suggests that the transmission of functional disorders within families could be attributed to genetic predispositions and shared environmental influences. The findings highlight the need for a more comprehensive understanding of the factors contributing to somatoform disorders, considering the biological underpinnings and the environmental contexts in which these disorders manifest. On the same leitmotiv, a recent study (Hur et al., 2019) conducted on a sample of 1,754 South Korean adolescent and young adult twins aimed to estimate the heritability of somatization and to explore the shared genetic and environmental influences between somatization and Hwabyung (HB), an anger syndrome. The sample included 367 monozygotic males, 173 dizygotic males, 681 monozygotic females, 274 dizygotic females, and 259 opposite-sex dizygotic twins. Participants completed self-report questionnaires on HB symptoms and somatization scales through telephone interviews.

The findings revealed that 43% of the variance in somatization was attributable to additive genetic factors. In comparison, the remaining 57% was due to individual-specific environmental influences, including measurement error, with no significant sex differences observed. A notable phenotypic correlation of 0.53 between HB and somatization was identified, suggesting a significant relationship. Bivariate model-fitting analyses indicated a genetic correlation of 0.68 between HB and somatization, signifying a substantial genetic overlap. However, the individual-specific environmental correlation, including correlated measurement errors, was 0.41, suggesting that while there is a shared genetic basis, the environmental factors contributing to the development of HB and somatization may be largely independent.

This study’s findings emphasize the complex interplay of genetic and environmental factors in the manifestation of somatization and HB, underscoring the importance of considering both in understanding and treating these conditions. The significant genetic correlation between the two suggests potential shared biological pathways, which could inform future research and treatment strategies. However, the distinct environmental influences highlight the role of individual experiences and contexts in developing these conditions, pointing to the need for personalized approaches in treatment and prevention.

Adult attachment styles (AAS) may play a significant role in further explaining the relationship between genetic and environmental factors. A recent fMRI study (Vrtička et al., 2012) delves into the modulation of social emotion perception and regulation by AAS. Conducted with 19 healthy adults, the research investigates how avoidant (AV) and anxious (AX) attachment styles affect neural responses to social emotions. AV individuals displayed increased activity in the dorsolateral prefrontal cortex (DLPFC) and left amygdala during cognitive reappraisal of unpleasant social stimuli, indicating a potential inefficiency in emotion regulation strategies. Additionally, during the suppression of positive social emotions, these individuals showed heightened activity in the supplementary motor area (SMA) and ventral caudate, suggesting a more significant effort to inhibit emotional expressions.

Conversely, AX individuals exhibited increased activation in the right amygdala and left parahippocampal cortex in response to negative and positive social stimuli, respectively, but only during spontaneous emotional judgments. This pattern suggests a heightened sensitivity to social cues without the corresponding difficulty in emotion regulation that AV individuals experience. The study’s findings underscore how AAS influences the neural underpinnings of social emotion regulation. Avoidant individuals may struggle with cognitive reappraisal and exhibit more significant neural effort during emotion suppression, while anxious individuals display heightened neural sensitivity to social stimuli.

In 2012, Vrticka and Vuilleumier conducted a comprehensive overview of the relationship between neuroscience, human social interactions, and adult attachment styles (AAS) (Vrtička and Vuilleumier, 2012). They offered a unique perspective on how individual differences in attachment impact emotional and social cognition through neurobiological processes. The authors recognized the critical role of attachment theory (AT) in understanding the development and maintenance of social bonds. They explored the neurobiological factors influencing attachment orientations and how they impact various social and emotional behaviors.

The review proposes a framework that combines current neuroscience with relational and attachment theories. This framework has two key components. The first component is a system that allows for rapid and automatic affective appraisals. This system encodes basic dimensions of safety versus threat or approach versus aversion tendencies in social contexts. It relies on limbic cortico-subcortical areas. The second component is a controlled processing system that engages in mentalizing processes such as theory of mind, self-reflection, and emotion regulation. This system uses frontotemporal areas.

This review explains how two different systems, called affective appraisal and emotional arousal, work together in social situations. These systems affect people differently, depending on their attachment styles. People with an avoidant attachment style tend to ignore or downplay positive social signals. Those with an anxious attachment style are more sensitive to negative cues, which can cause emotional arousal. These findings help us understand functional disorders, which are physical symptoms that do not have a clear medical cause. These disorders are affected by psychological factors, including how people process and regulate emotions (Edwards et al., 2012; Solms and Friston, 2018). Attachment styles play a significant role in emotional regulation and processing (see for example Liu and Ma, 2019; Eilert and Buchheim, 2023). People with an insecure attachment style may have trouble controlling their emotions (see for example Tammilehto, et al., 2022) and this may lead to experience physical symptoms which, in turn, could become predisposing factors for the functional disorders (Edwards, et al., 2012; Espay et al., 2018; Gilmour et al., 2020; Liang et al., 2021).

In conclusion, attachment styles can affect how people process and regulate emotions. This can lead to physical symptoms that are part, as triggers or predisposing factors, of functional disorders. Understanding this connection can help us develop better treatments for these disorders.

## Addictions and neuromodulation of depressive feelings

Addiction is a complex issue that arises due to the misuse of various substances, which triggers a specific type of brain plasticity that is common across all addictive substances. While we have a good understanding of the neurobiology of addiction, the exact role of certain neuromodulators, such as serotonin and dopamine, is still not entirely clear. Dopamine is the neurotransmitter that is most involved in the mechanisms of addiction.

Numerous medications can stimulate the release of dopamine in the nucleus accumbens, a crucial brain nucleus within the reward circuit. Typically, the prefrontal cortex receives a steady dopamine flow due to the ongoing activity of dopaminergic neurons that originate in the ventral tegmental area (VTA) and connect to the cortex. However, when an enriching experience or an extremely unpleasant event occurs, the dopaminergic neurons become much more active and fire rapidly. This sudden increase in activity causes a temporary but significant spike in dopamine levels. It is widely believed that these heightened dopamine levels during this rapid discharge phase are crucial for dopamine’s reward effect (Uhl et al., 2019; Becker-Krail et al., 2022).

The dopamine receptors (D1) stimulate both reward and conditioning mechanisms in which the amygdala, medial orbitofrontal cortex (OFC) and hippocampus are involved, thus enhancing memory and creating memories associated with substance use (Uhl et al., 2019). Memory processes allow the creation of an automatic association, i.e., a prediction of satisfaction (reward). In subjects with addiction, however, dopamine levels in the *nucleus accumbens* are lower than levels found in healthy subjects. This would be given by the tolerance/habituation mechanisms, which lead to behaviors (due to increased dopamine in the dorsolateral striatum) of habit and are no longer goal-directed, as in the early period of addiction (McSweeney et al., 2005; Lipton et al., 2019; Uhl et al., 2019). Over time, a change leads to goal-directed, habit behaviors, as the connections between the dorsolateral striatum and basolateral amygdala, which processes the cues that trigger habit behaviors, are reinforced (Lipton et al., 2019). Then, a reduced response of the reward system is generated. Concurrently, the absence of the psychoactive substance triggers adverse reactions, i.e., withdrawal (Uhl et al., 2019). The brain systems that regulate reward and stress responses are intimately connected. In the absence of the substance (e.g., opioids, alcohol or stimulants), a negatively perceived reaction is generated in the individual by the release of corticotropin-releasing factor (CRF) and dynorphin in the amygdala (Knoll and Carlezon, 2010; Zorrilla et al., 2014; Baumgartner et al., 2022). In turn, CRF stimulates the production of precisely Adrenocorticotropic Hormone (ACTH) in the pituitary gland, which stimulates cortisol production (Panksepp and Biven, 2012; Zorrilla et al., 2014; Uhl et al., 2019; Lightman et al., 2020). The hypothalamic–pituitary–adrenal axis coordinates neuroendocrine stress response systems (Herman et al., 2016).

A renewed abuse reduces stress-related reactions, ending the aversive reactions of withdrawal. However, there are alternative ways to reduce blood cortisol levels. It appears, for example, that serotonin may also play a role in inhibiting reward establishment and aversive states (Uhl et al., 2019).

This review emphasizes that oxytocin plays a role in reducing cortisol levels. First, studies show how dense the interactions between dopamine and oxytocin are in the context of social behaviors such as sex and pair bonding. The prefrontal cortex receives important oxytocin innervations, and receptors for dopamine and oxytocin are found in large populations in both the prefrontal cortex and nucleus accumbens. This evidence indicates how oxytocin plays a role in the expression of complex social behaviors and how, conversely, may shape such behaviors. In people with addiction, reduced dopamine receptors (due to prolonged overstimulation over time given by nerve pathways projecting from the VTA into the prefrontal cortex) resulting in less activation of these areas is partly responsible for impulsive and compulsive behaviors.

There exists a body of evidence which suggests that the neurotransmitters dopamine and oxytocin work in tandem to regulate addiction. Specifically, oxytocin is believed to facilitate abstinence while mitigating mechanisms of tolerance and dependence. This would be due to its relationship with dopamine: endogenous opioids in natural social situations control the expression of oxytocin neurons. Opioid-based drugs, therefore, act on oxytocin neurons by inhibiting them since they express opioid receptors. In addiction and abstinence, therefore, the oxytocin system, as well as the dopaminergic system, would be altered; this evidence prompts further investigation into the therapeutic role that OT might play in the treatment of addiction, particularly in the withdrawal phase (Baskerville and Douglas, 2010).

When managing stress, oxytocin is known for reducing stress-related reactions. Social support can also increase the production of oxytocin and lower cortisol levels. In addition, taking oxytocin directly through the nose has been shown to have an even more significant impact in reducing the neuroendocrine responsible for adverse stress reactions (Love, 2018). Recent findings show how oxytocin can reduce drug-seeking and drug-induced behaviors. This could be because oxytocin may have a direct role in glutamatergic pathways involved in addiction (Sundar et al., 2021). This evidence leads us to discuss the role oxytocin plays in prolonged and acute stress contexts.

## Neuromodulation of depression and anxiety

Interdisciplinary studies, as reviewed above, may help better understand many psychiatric problems. For instance, since social loss is one of the most robust precipitants of depression (Nelson and Panksepp, 1998) and oxytocin administration reduces PANIC/GRIEF, we could anticipate that oxytocinergic facilitators should be effective treatments for sadness and depression.

Postpartum depression is a clinical condition that is accompanied subjectively by conflictual ambivalent feelings of a mother toward her baby (Green, 1986; Lemaitre-Sillère and Bennett, 1998). Compared with non-lactating women, postpartum mothers (2 days after birth) who have received OT during labor (just after birth) have significantly lower scores on anxiety and aggression self-report scales and also score higher on a socialization scale, with the result remaining constant at 2 and 6 months postpartum (Jonas et al., 2008). Plasma OT and pregnancy OT plasma levels are very stable throughout all trimesters of pregnancy. They are positively associated with maternal–fetal attachment as measured by a prenatal attachment scale administered to the mothers (Levine et al., 2007). Feldman et al. (2007) analyzed OT levels in the first trimester and early postpartum period and found a positive correlation with certain maternal bonding behaviors, such as gazing at the infant, vocalizations to the infant, and affectionate touch. Of course, many measures of peripheral OT levels can be criticized since the correlation between central and peripheral oxytocin remains unclear in all human experiments, and the results are mixed in animal experiments. Nevertheless, it is worth noting that viewing images of one’s children activates dopaminergic pathways in the mothers’ brains associated with reward, where there are also high levels of OT receptors (Bartels and Zeki, 2004).

The findings are consistent with clinical studies showing an association between specific adult attachment styles and depression (Carnelley et al., 1994). In particular, of the four categories of attachment style, only insecure attachment is linked with the risk of developing depression (Mickelson et al., 1997; Murphy and Bates, 1997). Recent studies suggest interactions between central OT and attachment style (Bartz J. A. et al., 2010; Rockliff et al., 2011). For instance, Bartz and colleagues found that intranasal *OT promotes the recollection of negative representation of the mother in individuals with insecure attachment styles*, with opposite effects in individuals with secure attachment styles (Bartz J. et al., 2010). These findings contrast the hypothesis that oxytocin would increase positive feelings (Yong, 2012).

On the other hand, a possible interpretation of these findings would be that OT facilitates the disclosure of one’s representations by enhancing a basic feeling of safety and comfort. This evidence is in line with our hypothesis that *OT may be an essential neuromodulator of a homeostatic system related to safety conditions* reflecting the experience by the subject, without distinction of the fact these same experiences were positive or negative in terms of wellbeing. As well known in clinical practice, the feeling of safety is the prerequisite for sharing personal experiences and representing oneself and others. Indeed, considering that safety cuts across both social and non-social domains, this primal “common denominator” may help explain various paradoxical and fragile specific social effects seen with oxytocin (Bartz et al., 2011). Modern anti-depressants have been employed in postpartum depression. Now that there are ways of administering exogenous OT via intranasal airways, which are medically approved in Europe, have no known addiction liability, and seem to be fundamentally free of side effects, there is a role for off-label use of neuropeptides in treating this type of depressions, with positive effects that can occur promptly as opposed to over several weeks of treatment with a classical antidepressant. Our group has started the evaluation of whether intranasal administration of oxytocin can alleviate feelings of despair in those mothers who experience depression soon after the birth of their children. Such a project, in the context of psychoanalytic therapy, has been initiated (Ozkarar and Clarici, 2008; Pellizzoni et al., 2009; Clarici and Pellizzoni, 2013) with preliminary results showing a facilitatory role of OT in maintaining a positive therapeutic alliance in the course of a dynamically oriented psychotherapy in postpartum depressed mothers (Clarici et al., 2015). OT’s pharmacologic properties make it most attractive as a potential antidepressant drug in postpartum depression, with the main aim of improving the quality of infant-mother interaction through natural endogenous substances and psychosocial interventions.

In depressed patients, the hyperactivation of HPA axis, and consequently, high cortisol level, is well established. Additionally, plasma oxytocin is negatively associated with scores of depression symptoms (Scantamburlo et al., 2007; Cyranowski et al., 2008). Central OT administration has been shown to have anxiolytic properties on endocrine and behavioral systems. This seems to be due to the effect of the neuropeptide on the HPA system (Windle et al., 1997; Bale et al., 2001). However, it is still unclear if it has a direct effect on the axis or an indirect effect through other structures, such as the *bed nucleus of the stria terminalis* (Dunn, 1987) or the amygdala (Dunn and Berridge, 1990). OT does not decrease salivary cortisol levels in men with a history of early parental separation as in control males, indicating a possible abnormal HPA response to OT in these subjects (Meinlschmidt and Heim, 2007). This study confirms a relation between the attachment system and possible dysregulation of these neural systems due to a premature loss experience. OT may exert its effects by directly inhibiting the amygdala (Davis, 1992; Adolphs et al., 1995). Further, recent contributions show that long-term anxiety and depressive states associated with development are associated with greater amygdala volume (van Elst et al., 2003). In the laboratory, the amygdala activation is reduced by intranasal administration of OT (Dębiec, 2005).

From this perspective, the role of the amygdala in major depressive and anxiety disorder is most interesting (Pezawas and Meyer-Lindenberg, 2005). Functional imaging studies observed an increased metabolism in the amygdala in subjects with major depressive disorder (Fales et al., 2009). Related to this, studies have shown that OT reduces both social anxiety (Heinrichs and Domes, 2008) and amygdala responses to fearful faces (Kirsch et al., 2008), implying a possible trade-off of OT and amygdala responsivity and, in turn, a sensation to trust the other and to create a bond with people and possibly offspring.

## Conclusion

This review delves into the intricate workings of various neuromodulators in regulating various physiological and psychological functions, including safety, social bonding, and affective processes. The paper pays particular attention to how the neuromodulator oxytocin impacts attachment and prosocial behaviors, providing in-depth insights into how this hormone shapes our relationships and emotional experiences in normal development and psychopathology. Panksepp’s work is cited to emphasize the connection between oxytocin and feelings of safety, suggesting that oxytocin and other neuromodulators play a crucial role in establishing an affective background of safety and security. The focus is on the oxytocin system’s involvement in various aspects of human development, from parent-infant attachment to adult relationships, suggesting a standard regulatory homeostatic system. The discussion extends to the overlap between oxytocin and dopamine systems, highlighting their implications for motivational states and affiliative behavior. Studies have shown that the rise in oxytocin levels in the brain can improve psycho-relational behaviors. This is believed to be due to the release of a primary affective signal that enhances comfort, which is associated with the fundamental feeling of safety. As a result, individuals experience a boost in social confidence and a decrease in feelings of panic or grief. Essentially, the release of oxytocin triggers a cascade of positive emotional responses that can lead to beneficial outcomes for individuals in social situations. The oxytocinergic system plays a crucial role in regulating emotions and behaviors such as trust, social cognition, empathy, love, and compassion. It has been observed that changes in the regulatory processes involving oxytocin and various neurotransmitters connected to it can contribute to the development and persistence of depressive disorders. It is important to note that this response should not be regarded solely as a disease, but also as an adaptive mechanism of the brain in response to predictive and false inferences that trigger depressive symptoms. One notable sentence that helps connect psychopathology with the predictive coding theories of Friston (2023) is as follows:

The narrative starts with the simple premise that if psychology— read as belief updating in the brain—can be cast as a computational process of inference, it follows that *psychopathology just is false inference*. [page 257]

The role of oxytocin is connected to social bonding, postpartum depression and the interrelation between social and physical pain systems, with evolutionary and clinical aspects. It is a potential modulator of affective and maternal urges, affecting emotional responses to panic and grief more strongly than physical pain. Even at high doses, oxytocin’s effects are subtle, but it is involved in maintaining emotional homeostasis. Overall, this review confirms that oxytocin plays a multifaceted role in regulating emotions, social behaviors, and mental well-being throughout various stages of development.

## Author contributions

AC: Conceptualization, Writing – original draft, Writing – review & editing. MB: Conceptualization, Writing – review & editing. YR: Writing – review & editing. JP: Conceptualization, Methodology, Writing – review & editing.

## In memoriam

This paper was written with the mentorship and editing by late professor Jaak Panksepp, and it is one of the last works before he passed away, leaving the scientific community with a deep feeling of loss. Therefore, this work suffers from the loss of the scientist who, among us authors, possessed the vastest knowledge of the brain mechanisms of neuromodulation. We believe that, even if this work has inevitably been affected by this loss, we trust that Jaak Panksepp would have approved the hypotheses on the neuromodulatory processes, which, based on his guidance, have led us to extend his conclusions to the psychiatric, psychotherapeutic and psychopathological fields. The publication is thus posthumous (one of the authors, AC obtained written permission from Prof. Panksepp’s wife, Anesa Miller, anesam98@gmail.com), to maintain his name as one of the authors, as a sign and homage of our indebtment to Jaak Panksepp for his great wisdom, vast knowledge, that he perfused to us with profound intellectual and scientific honesty.

## Footnotes

^1^It should be noted that in psychiatry, for several decades now, as it is well-known, it has become common practice to refer to mental illness using the term “psychiatric disorder” (e.g., as in bipolar disorder, obsessive-compulsive disorder, or personality disorder, etc.), a term which is evocative of an entropic tendency affecting the psyche.

^2^As challenging as it may appear for a reader with a psychological background to delve into these arguments drawn from theoretical physics, we want to emphasize (as we have extensively done in the mentioned article; Clarici et al., 2019) that this principle may be considered a conceptual corollary of the equally well-known principle of *intrapsychic safety* put forth by Sandler (in the 1960s) in the context of psychopathology. Similarly, it aligns this Safety principle with the fundamental need for a secure base present in the infant (as in many animal species) in the field of intersubjective attachment development, as proposed by Bowlby and his followers (also in the same decade of the 20th century – i.e., the 60ies).

^3^Freud in the Outline of Psychoanalysis (1938 [1940]) states: “Just as the id is directed exclusively to obtaining pleasure, so the ego is governed by considerations of safety. The ego has set itself the task of self-preservation, which the id appears to neglect” (p. 199).
